# Developmental trajectory of transmission speed in the human brain

**DOI:** 10.1038/s41593-023-01272-0

**Published:** 2023-03-09

**Authors:** Dorien van Blooijs, Max A. van den Boom, Jaap F. van der Aar, Geertjan M. Huiskamp, Giulio Castegnaro, Matteo Demuru, Willemiek J. E. M. Zweiphenning, Pieter van Eijsden, Kai J. Miller, Frans S. S. Leijten, Dora Hermes

**Affiliations:** 1 Department of Physiology and Biomedical Engineering, Mayo Clinic, Rochester, MN USA; 2 Department of Neurology and Neurosurgery, UMC Utrecht Brain Center, University Medical Center Utrecht, Utrecht, the Netherlands; 3 Stichting Epilepsie Instellingen Nederland (SEIN), Zwolle, the Netherlands; 4 Department of Neurosurgery, Mayo Clinic, Rochester, MN USA

**Keywords:** Cognitive ageing, Neurophysiology, Databases, Development of the nervous system

## Abstract

The structure of the human connectome develops from childhood throughout adolescence to middle age, but how these structural changes affect the speed of neuronal signaling is not well described. In 74 subjects, we measured the latency of cortico-cortical evoked responses across association and U-fibers and calculated their corresponding transmission speeds. Decreases in conduction delays until at least 30 years show that the speed of neuronal communication develops well into adulthood.

## Abstract (web summary)

This study mapped the developmental trajectory of transmission speed in the human brain by using electrical pulses and intracranial recordings. The authors found that these pulses travel with increasing speeds up to at least the age of 30.

## Main

The development of rapid communication between human brain regions is essential for cognitive function. The speed of neuronal transmission is fundamental to the temporal organization of neuronal activity^1^ and is a core component in many computational human brain models^2^. The developing axons in the human brain support rapid neuronal transmission, influencing whether electrical signals arrive at the same or at different times and shaping the timescales of functional connectivity^3^. However, little is known about the maturation process of transmission speed in the human brain, partially because the axonal diameter in the adult human brain is relatively large compared with most other mammalian species^4^.

Anatomical studies indicate that the structural human connectome follows a long developmental trajectory: postmortem studies have shown that myelination starts in the late prenatal period and continues into late adolescence^5^. Magnetic resonance imaging (MRI) analyses have demonstrated that white matter properties change across the life-span^6^, often reaching a plateau around 30 years of age.

However, electroencephalography and magnetoencephalography studies that approximate transmission speed by measuring the latency of visual evoked potentials, show highly variable ages at which development plateaus. While studies consistently find decreases in the latency of the visual evoked potential at around 100 ms during infancy and early childhood (<13 years)^7–9^, the developmental plateau at which latency decreases change to latency increases differs across studies. Some studies report that evoked potential latency starts increasing after age 13 (ref. ^10^), others report no change in latency during adolescence^11,12^, others report that latency decreases up to age 20 followed by an increase^13–15^, while others report that latency decreases up to age 40 years^16,17^ (Supplementary Table 2). One cortico-cortical evoked potential (CCEP) study reported that conduction delays in subjects older than 15 years were only 1 ms faster compared with younger subjects^18^. This poses the question of whether the long structural maturation process translates to changes in neuronal transmission speed.

**Fig. 1:**
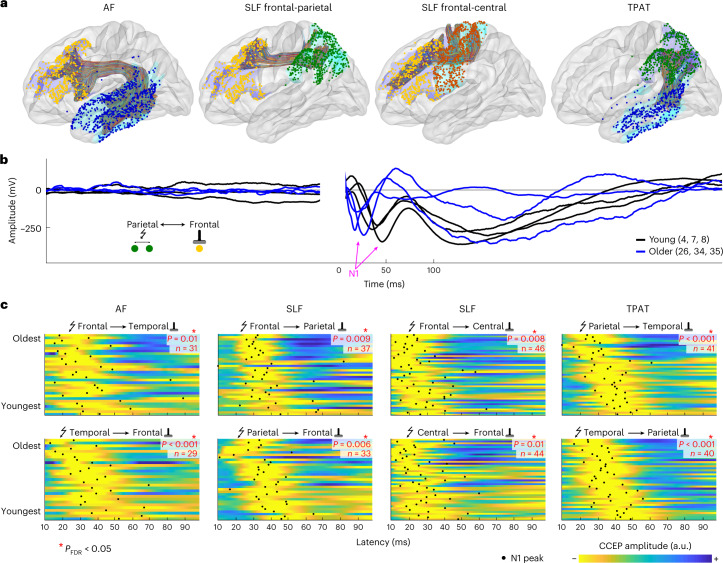
Electrode positions, fiber tracts and evoked potentials. **a**, MNI brain surface showing white matter tracts and electrode positions at endpoints from all 74 subjects. **b**, CCEPs from young subjects (black lines, 4, 7 and 8 years old) and older subjects (blue lines, 26, 34 and 35 years old) across the SLF frontal-parietal tract after parietal stimulation. The N1 peak is indicated by a magenta arrow. **c**, CCEP responses for all subjects and their N1 peak latency (black dots), organized by age for each white matter tract and direction. CCEPs are unit length normalized and yellow indicates the largest negative deflection. A red asterisk indicates a significant negative correlation between age and N1 latency (Spearman’s *ρ*, two-sided, *P* < 0.05, FDR correction for multiple comparisons). The statistical values from left to right, top to bottom are: *ρ* = −0.43, *P* = 0.01, *n* = 31; *ρ* = −0.43, *P* = 0.009, *n* = 37; *ρ* = −0.40, *P* = 0.008, *n* = 46; *ρ* = −.64, *P* < 0.001, *n* = 41; *ρ* = −0.62, *P* < 0.001, *n* = 29; *ρ* = −0.48, *P* = 0.006, n = 33; *ρ* = −0.37, *P* = 0.01, *n* = 44; *ρ* = −0.61, *P* < 0.001, *n* = 40.

To characterize the maturation process of transmission speed in the human brain, we measured single-pulse-stimulation-evoked CCEPs during human intracranial electrocorticography (ECoG) recordings in a large group of 74 subjects aged 4–51 years old. CCEPs often show an early surface negative deflection (N1) within 100 ms after stimulating another electrode pair. Figure 1b shows an example of how the N1 response measured in frontal areas upon parietal stimulation peaks around 45 ms in three young subjects (aged 4, 7 and 8 years), while peaking around 1.5–2 times faster—around 25–30 ms—in three older subjects (aged 26, 34 and 35 years).

This rapid negative N1 potential measured with ECoG on the brain surface has been related to direct cortico-cortical white matter connections^19^, and is thought to be generated by synchronized, excitatory synaptic activation of the distal layer apical dendrites of the pyramidal cells^20^. While this feature selection likely ignores many other aspects of the evoked potential that provide a richer characterization of cortico-cortical communication^21^, the N1 response provides insight into transmission speed across several bundles in the human white matter connectome^18,22^.

To quantify age-related changes in conduction delays across some well described association fiber bundles, we use a white matter atlas to extract CCEPs across the arcuate fasciculus (AF), two sections of superior longitudinal fasciculus (SLF) and the temporo-parietal aslant tract (TPAT) in each subject^23^ (Fig. 1a). The SLF was segmented into frontal-parietal and frontal-central connections given the different lengths of these segments. We find that N1 latency correlates negatively with age across all four pathways (Fig. 1c; Spearman’s *ρ*, *P*_FDR_ < 0.05). We note that the number of CCEPs does not change consistently with age, indicating no age-related changes in the overall level of connectivity (Supplementary Fig. 2). The latency decreases show that conduction delays across association fibers in the human brain decrease with development.

**Fig. 2:**
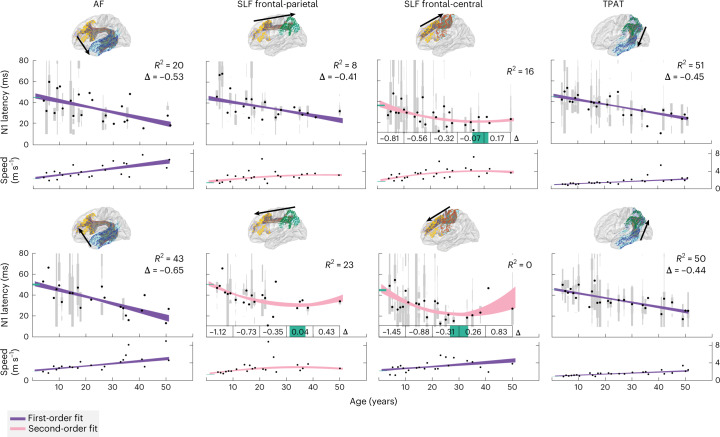
Developmental trajectory of conduction delay and speed across long-range connections. Average transmission latency and speed estimated by the N1 component for the AF, frontal-parietal SLF, frontal-central SLF and TPAT (left to right). Gray bars show distributions within each subject, the bar width scales with the number of measured responses. Black dots show N1 latency or speed averaged across subjects of the same age. First- and second-order polynomial models (fit with robust regression and shown with 95% confidence intervals) explain the changes in N1 latency or speed as a function of age. The coefficient of determination (*R*^2^) indicates the variance in latency explained by age (compared with a mean latency rather than a zero baseline). The *R*^2^ is calculated with leave-one-out cross-validation and used to indicate whether the first-order (purple) or second-order (pink) polynomial model explained more variance in the data. For second-order polynomial model fits, the 95% confidence interval is shown in green for the minimum age on the *x* axis and for the N1 latency intercept on the *y* axis. For the first-order polynomial fits, insets show the slope change (*Δ*) in milliseconds per year. For the second-order polynomial fits, the slope change is displayed in milliseconds per year averaged across 10 years of age. The sample sizes (*n* = number of ages) for the top row are: 23, 23, 27 and 26 (from left to right), and 21, 22, 23 and 26 (from left to right) for the bottom row.

We then describe the maturation process across these association fibers by fitting a first- and second-order polynomial model where age predicts N1 latency (Fig. 2). These models have been used before in MRI studies of development^6,24^. A robust regression and leave-one-out cross-validation further ensures that single subjects do not drive the results and lets the data indicate which connections are better described by a linear or quadratic model. N1 latency is well predicted by age in the AF, frontal-parietal SLF, frontal to central SLF and TPAT. Moreover, conduction delays mature well into adulthood. Before the age of 10 years, latency decreases by around 0.73 ms per year on average, while between age 20 and 30 years, latency decreases less rapidly by around 0.43 ms per year on average. The quadratic models indicate that a minimum latency of around 25 ms was reached after age 30 years. These small, yearly changes in conduction delays translate in an increase in transmission speed from childhood (6–13 years) to adulthood (19–64 years) of around twofold from roughly 1.5–3 m s^–1^ to 3–6 m s^–1^ (Fig. 2). This indicates that the development of rapid transmission speed across long-range association fibers matures well throughout adolescence.

**Fig. 3:**
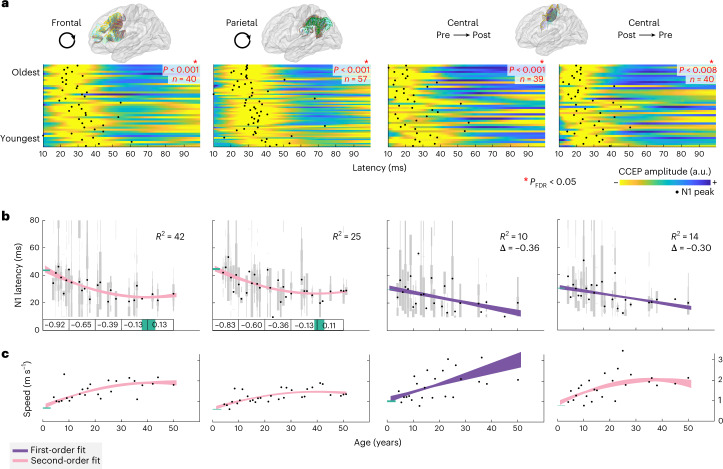
Short-range connections decrease in conduction delay with age. **a**, The CCEP responses and their N1 peak latencies (black dots) ordered by age for atlas-based U-fiber connections on frontal, parietal, pre- to postcentral and post- to precentral regions. CCEPs are unit length normalized and yellow indicates the largest negative deflection. A red asterisk indicates a significant negative correlation between age and N1 latency (Spearman’s *ρ*, two-sided test, FDR-corrected, *P*_FDR_ < 0.05). The statistical values from left to right and the number of subjects *n* are: *ρ* = −0.55, *P* < 0.001, *n* = 40; *ρ* = −0.53, *P* < 0.001, *n* = 57; *ρ* = −0.53, *P* < 0.001, *n* = 39; *ρ* = −0.43, *P* = 0.008, *n* = 40. **b**, Average conduction delays estimated by the N1 latency. Gray bars show distributions within each subject, bar width scales with the number of measured responses. Black dots show N1 latency averaged across subjects of the same age. First- and second-order polynomial models (shown with 95% confidence interval) explain the changes in N1 latency as a function of age. The sample sizes (number of ages) are 25, 32, 25 and 25 from left to right. Explained variance (*R*^2^) calculated with leave-one-out cross-validation indicates whether the first-order (purple) or second-order (pink) polynomial model explains more variance. For all model fits, the 95% confidence interval of the N1 latency intercept (latency at the youngest age) is shown in green on the *y* axis. For the first-order polynomial fits, insets show the slope change (*Δ*) in milliseconds per year. For second-order polynomial model fits, the 95% confidence interval of the minimum age is shown in green on the *x* axis and the slope change is displayed in milliseconds per year averaged across 10 years of age. **c**, Same as **b** for transmission speed based on the average U-fiber length (m s^–1^) and the same sample sizes.

Short-range connections across neighboring gyri such as the pre- and postcentral gyrus and within frontal and parietal regions are supported by U-fibers. Latencies decrease significantly with age across these short connections (Fig. 3a,b), with corresponding increases in speed (Fig. 3c). U-fibers overall reached speeds up to around 2 m s^–1^. The model fits show that latencies decrease until age 35 years or older, indicating that transmission speeds across U-fibers mature well throughout adolescence. Interestingly, the frontal and parietal U-fibers had longer latencies during early childhood (>40 ms) compared with the central U-fibers. This is consistent with the idea that sensorimotor regions mature before frontal and parietal association areas^25^.

While the overall developmental trajectory of the U-fibers was comparable with that of association fibers there were also important differences. The latencies across the longest association fibers (AF and parietal-frontal SLF) during childhood range from around 45 to 55 ms (Fig. 2), while the childhood latencies of central U-fibers range from around 30 to 40 ms (Fig. 3b). However, at adulthood, latencies of 20–30 ms are typical for both association and central U-fibers. The maximum speeds reached across the U-fibers (around 2–3 m s^–1^) are therefore smaller compared with the longer range association fibers (around 3–6 m s^–1^). Axon diameters show large variations ranging from 0.16 to 9 µm in the human brain and, given the limitations of the cranial space, only a small number of large axons can have a larger diameter^1,4^. In myelinated axons, the conduction velocity increases approximately linearly with axon diameter^26^. Smaller U-fiber axons compared with larger association fiber axons may explain the slower speeds in the U-fibers.

The data reveal variability within and between the subjects. Some variability can probably be attributed to a heterogeneous subject population with different axonal properties and noise levels. Other variability may be explained by the fact that, in many natural processes, increases in the mean are related to increases in variability (such as firing rates typically following a Poisson distribution^27^). We indeed find that slower N1 responses often had increased variance (Supplementary Fig. 4) and increased widths (Supplementary Fig. 5), while we found no evidence for a relation between subject’s age and variance in latency (Supplementary Fig. 3). This indicates that faster cortico-cortical connections allow for overall more precise timing, whereas timing is less precise in slower cortico-cortical connections.

Our data indicate that transmission speeds are still maturing during adolescence and early adulthood. Many psychopathologies, like schizophrenia, anxiety disorders, depression and bipolar disorders, can emerge during these periods^28^, emphasizing the potential importance of our findings for these diseases. We note that, while our subjects suffered from epilepsy, there were no consistent effects of the seizure onset region on latency (Supplementary Figs. 6 and 7), and epilepsy may merely have added noise to the estimates. The large number of subjects allows us to establish a normative baseline with which different pathologies may be compared.

A long maturation process of transmission speed aligns with findings from noninvasive neuroimaging studies that show that association white matter pathways in the human brain mature well into early adulthood^24,29,30^. MRI studies of the white matter pathways have captured some of these processes and show that white matter development follows a quadratic function with a peak between 30 and 40 years of age^6,31^. This trajectory is comparable with the developmental trajectory of conduction delay that is shown in our data. While this long developmental trajectory is consistent with some evoked potential studies^16,17^, other early sensory evoked potentials may show a much faster developmental trajectory until the age of about 20 years^10,13–15^. Some of the variability between evoked potential studies may stem from the development of intermediate synapses between the sensory input and brain measurements. Alternatively, the fast development of some early sensory evoked potentials could also be related to the fact that projection fibers to sensory regions develop faster compared with association fibers^24,29^. Sensory evoked potentials that spread across projection fibers to sensory regions may mature more rapidly compared with the stimulation-evoked potentials across the association fibers measured in the current study.

A simple characterization of the timing of direct cortico-cortical interactions has large implications for the temporal dynamics of brain function. Neuronal synchrony depends on the precise timing, and development can therefore either benefit or deteriorate synchronized brain activity^1^. Twofold increases in the speed of transmission were observed in long-range as well as short-range connections in the human brain. The large, consistent effects of age on transmission speed in our measurements provide normative estimates for the timescales of cortico-cortical signaling in distributed as well as local human brain networks.

## Methods

### Subjects

All subjects who underwent epilepsy surgery in the University Medical Center (UMC) Utrecht between 2008 and 2020 were included in a retrospective epilepsy surgery database^32^, with approval of the Medical Research Ethical Committee of UMC Utrecht. For subjects included between January 2008 and December 2017, the Medical Research Ethical Committee waived the need for informed consent. Since January 2018, we explicitly ask subjects for informed consent to collect their data for research purposes. No statistical methods were used to predetermine sample sizes and we included all subjects who underwent single-pulse electrical stimulation for clinical purposes during the intracranial grid monitoring period between 2012 and 2020 and met inclusion criteria. Subjects were not provided with compensation. In total, 74 subjects were included in this study (median age 17 years (4–51 years), 38 females), thus spanning age ranges from childhood (6–13 years), adolescence (14–18 years), young adult (19–33 years) and middle age (49–64 years)^33^. Inclusion criteria were the absence of large brain lesions and that electrode positions could be determined based on a computed tomography scan coregistered with a T1 MRI^34^. After electrode localization, electrodes were labeled according to the Freesurfer based Destrieux atlas segmentation^35,36^. The electrodes were well distributed across the age groups (Supplementary Fig. 9). For visualization, the individual subject’s electrode positions were converted to Montreal Neurological Institute (MNI)152 space. During the evaluation for epilepsy, the seizure onset zone and eloquent cortex are delineated and a resection area suggested to the surgeon. No different experimental conditions were applied to the subjects and randomization was not possible. Data collection and analysis were not performed blind to the conditions of the experiments.

All CCEPs were reviewed and 4 runs with incorrect stimulation onsets were removed. Furthermore, electrodes that overlapped with another grid, were located on small structural abnormalities or had excessive noise were excluded from analyses. On average, across all subjects, 6.3% of electrodes were excluded. We additionally excluded stimulation pairs that introduced baseline offsets on many measured channels. To ensure that the epilepsy did not affect the result in a systematic manner, the seizure onset zone was annotated in 30 subjects by a clinical neurophysiologist. This allowed comparison of latencies in and outside of the seizure onset region (Supplementary Figs. 6 and 7).

### Acquisition

Long-term ECoG data were recorded with subdural electrode grids and strips of 4.2 mm^2^ contact surface and an interelectrode distance of 1 cm (Ad-Tech and PMT). Additional depth electrodes were implanted in several subjects but are not included in analyses because they were typically placed in lesions visible on an MRI. Single-pulse electrical stimulation was performed during ECoG recordings with data sampled at 2,048 Hz using a MicroMed LTM64/128 express electroencephalography headbox with integrated programmable stimulator (MicroMed). The stimulation onset was determined accurately by MicroMed hardware, but we note that electrical stimulation creates an artifact from about −9 ms to 9 ms around stimulation onset as channels are coupled to the ground during stimulation. Ten monophasic stimuli with a pulse width of 1 ms were applied at a frequency of 0.2 Hz to two adjacent electrodes. Polarity was alternated after five pulses in 27 of the subjects such that stimulation artifacts are reduced by averaging. A current intensity of 8 mA was used, but in case electrodes were located near central nerves or in the primary sensorimotor cortex, the intensity was lowered to 4 mA to avoid pain or twitches. Changes in amplitude did not systematically influence the results (Supplementary Fig. 1).

### N1 latency calculation

To estimate conduction delays across different connections, we calculated the latency of the earliest surface negative deflection in the CCEP in 9–100 ms after stimulation. This response is also referred to as the N1 and is thought to be generated by synchronized, excitatory synaptic activation of the distal layer apical dendrites of the pyramidal cells^20,22^ and spread through white matter^19,37^. For each electrode, ten epochs with a time window of 2 s prestimulus to 3 s poststimulus, time-locked to the stimulus, are corrected for baseline (median signal in a time window of 900 ms before stimulation (−1 s to −0.1 s) and averaged for each stimulus pair^38^. For each averaged epoch, the median is subtracted (−2 s to −0.1 s), and the s.d. is calculated in this prestimulus window. N1s are detected when the evoked response exceeds 3.4 × s.d. in a time window of 9–100 ms poststimulation, excluding earlier times due to potential stimulation artifacts.

Stimulation artifacts can potentially spread to nearby electrodes through volume conduction and the following helped ensure that this did not affect our results. First, volume conduction effects are largest in the first 1–8 ms after electrical stimulation^39^, and N1 detection was done after this time, from 9 to 100 ms. Second, we excluded electrodes within 13 mm from the stimulated electrode pair, at which distance the effects of volume conduction are largely negligible^40^. Lastly, in a previous manuscript using a subset of these data, we ensured that volume conduction did not play an important role by showing that the latencies differ across measured electrodes for a single stimulated pair^41^. We apply a similar method and show in Supplementary Fig. 8 that the detected N1 latencies varied across measured electrodes.

While the CCEP waveform has more complex features, the N1 component is the most robust and relevant feature to answer questions about direct electrical conduction^18^. The N1 is measured robustly with ECoG at the brain surface and can be detected as early as 10 ms after stimulation onset. The N1 has been related to direct cortico-cortical connections in many other CCEP studies of, for example, the motor system^42^, cingulum bundle^43,44^, frontal aslant tract^45^ and the superior longitudinal fasciculus^46^. Moreover, previous studies showed that the N1 corresponds relatively well with diffusion-MRI-derived white matter endpoints^47^, the N1 latency relates linearly with the distance traveled along a fiber bundle^48,49^ and that the N1 propagation velocity correlates with fractional anisotropy in the white matter^37^.

### Integrating electrode locations with a white matter atlas

The connectivity between the frontal, temporal, parietal and pre/postcentral (primary sensorimotor) areas was investigated based on the AF, the SLF and TPAT. We focus on these connections, and exclude connections to regions without sufficient electrode coverage for across-subject correlations, such as the occipital lobe. Each of these tracts was defined based on the population-averaged tractography atlases HCP1065 (AF, SLF, TPAT)^23^ and HCP842 (U-fibers)^50^. The SLF was split into two sections connecting frontal and parietal and frontal and central brain regions, because merging these sections would lead to inaccurate estimates of the length of the SLF and bias transmission speed estimates described in the next section. We subsequently matched the ECoG electrodes, located on the gray matter surface, to the tractography atlas using the gray matter endpoint probability estimates of the tracts^23^. In this way, we were able to investigate the CCEP based connectivity for different fiber tracts.

### Transmission speed estimation

To estimate the transmission speed along the tracts, we calculated the tract length in each subject. Using ANTs registration implemented in lead-dbs^51^ between the subject MRI and MNI space, the tracts from the atlases were registered to the native space of each subject. In each subject’s native space, the length of each tract was then calculated by taking the average length over all tract fibers in native space. To estimate transmission speed, the latency of each CCEP along a specific tract was divided by the respective length of the tract to obtain a speed in meters per second.

### Statistics

To describe the relationship between age and conduction delay and/or age and transmission speed, we fit a first- and second-order polynomial model where age predicts the N1 latency or the transmission speed. These models have been used before in MRI studies to characterize development-related changes in gray and white matter properties^6,52^. Fitting these models with leave-one-out cross-validation lets the data indicate whether the development of different connections is better described by a linear model or a quadratic model with a local minimum. To ensure that certain datapoints with high leverage did not unduly influence the results, we performed a robust regression with bisquare weight function and a tuning constant of 4.685. Data distribution was assumed to be normal but this was not formally tested. The coefficient of determination (*R*^2^) was used to indicate how well the model described the data:

$$R^2 = 1 - \frac{{\mathrm{SS}_{\mathrm{res}}}}{{\mathrm{SS}_{\mathrm{tot}}}}$$

in which

$$\mathrm{SS}_{\mathrm{res}} = \mathop {\sum}\limits_i {\left( {y_i - f_i} \right)^2} \,{{{\mathrm{and}}}}\,\mathrm{SS}_{\mathrm{tot}} = \mathop {\sum}\limits_i {\left( {y_i - \overline y } \right)^2}.$$

We note that the *R*^2^ provides the explained variance relative to a baseline model that predicts the average $\overline y$. If the model predicts the data better than baseline, *R*^2^ will be larger than 0, if the model predicts the data worse than baseline, *R*^2^ can be smaller than 0. The *R*^2^ therefore indicates how much of the variance in latency is predicted by age as compared with no change with age. When necessary, statistical tests were corrected for multiple comparisons using a false discovery rate (FDR) correction.

### Reporting summary

Further information on research design is available in the Nature Portfolio Reporting Summary linked to this article.

## Online content

Any methods, additional references, Nature Portfolio reporting summaries, source data, extended data, supplementary information, acknowledgements, peer review information; details of author contributions and competing interests; and statements of data and code availability are available at 10.1038/s41593-023-01272-0.

## Supplementary information

Supplementary Information Supplemental Figs. 1–9 and Tables 1 and 2.

Reporting Summary

## Data Availability

The data that support the findings of this study are being made available in BIDS format on OpenNeuro: https://openneuro.org/datasets/ds004080. Atlases of white matter tracts were defined based on the population-averaged tractography atlases HCP1065 (AF, SLF, TPAT)^23^ and HCP842 (U-fibers)^50^: https://brain.labsolver.org/hcp_trk_atlas.

## Code availability

The code to analyze the data and generate all figures of this manuscript is available on GitHub: https://github.com/MultimodalNeuroimagingLab/mnl_ccepBids.
