# Supplementary material for: Developmental trajectory of transmission speed in the human brain
Source: Nat Neurosci. 2023 Mar 9;26(4):537–41. doi: 10.1038/s41593-023-01272-0 (PMC10076215; doi:10.1038/s41593-023-01272-0)
Supplement: Supplementary file 1 — Supplemental Figs. 1–9 and Tables 1 and 2. [file 41593_2023_1272_MOESM1_ESM.pdf]

nature neuroscience

Brief Communication

https://doi.org/10.1038/s41593-023-01272-0

Developmental trajectory of transmission speed in the human brain

In the format provided by the authors and unedited

Supplementary Materials for

Developmental trajectory of transmission speed in the human brain

Dorien van Blooijs[1,2,3†], Max A. van den Boom[1,4†], Jaap F. van der Aar[2], Geertjan M. Huiskamp[2], Giulio Castegnaro[2], Matteo Demuru[2], Willemiek J.E.M. Zweiphenning[2], Pieter van Eijsden[2], Kai J. Miller[4], Frans S.S. Leijten[2], Dora Hermes[1]*

[1] Department of Physiology and Biomedical Engineering, Mayo Clinic; Rochester, MN, USA

[2] Department of Neurology and Neurosurgery, UMC Utrecht Brain CenterBrain Center Rudolf Magnus Institute of Neuroscience, University Medical Center Utrecht; Utrecht University, The Netherlands

[3] Stichting Epilepsie Instellingen Nederland (SEIN); Zwolle, The Netherlands

[4] Department of Neurosurgery, Mayo Clinic; Rochester, MN, USA

[†] Authors contributed equally

*Correspondence: hermes.dora@mayo.edu

This PDF file includes:
Supplementary Figures 1 to 9
Supplementary Tables 1 and 2

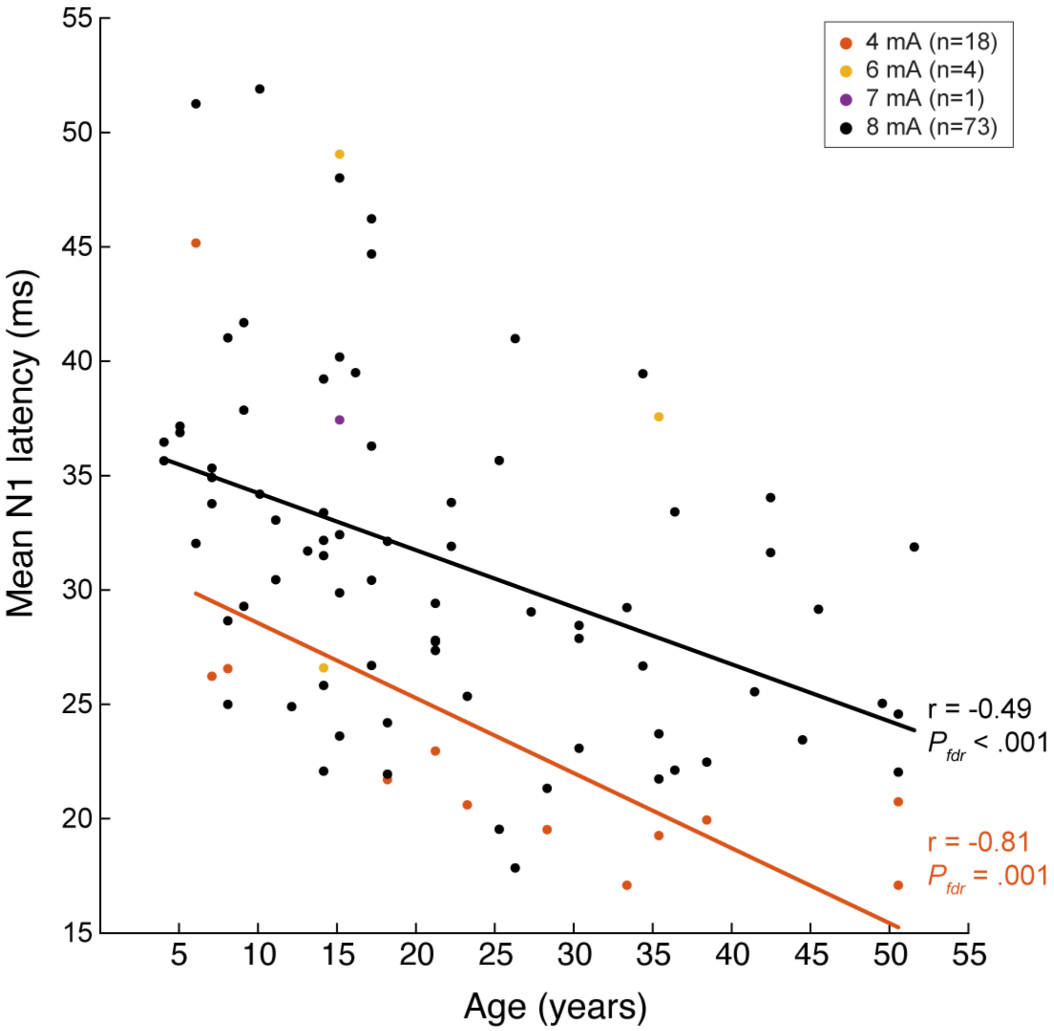

Supplementary Figure 1. Similar relation between age (years) and N1 latency (ms) at different stimulation currents. To ensure that the relation between age and latency was not driven by the fact that some electrode pairs were stimulated with a current of 8 mA, while others had a current of 4, 6 or 7 mA, we calculated the average latency per subject across all connected electrodes. The relation between age and latency across all subjects shows a significant (two-sided Spearman's ρ, $P_{\text{FDR}} < .05$) negative relation between age and latency for stimulation with both 4 mA (P_{FDR} =.001) and 8 mA (P_{FDR} <.001). 6 mA and 7 mA had too few samples for a robust fit.

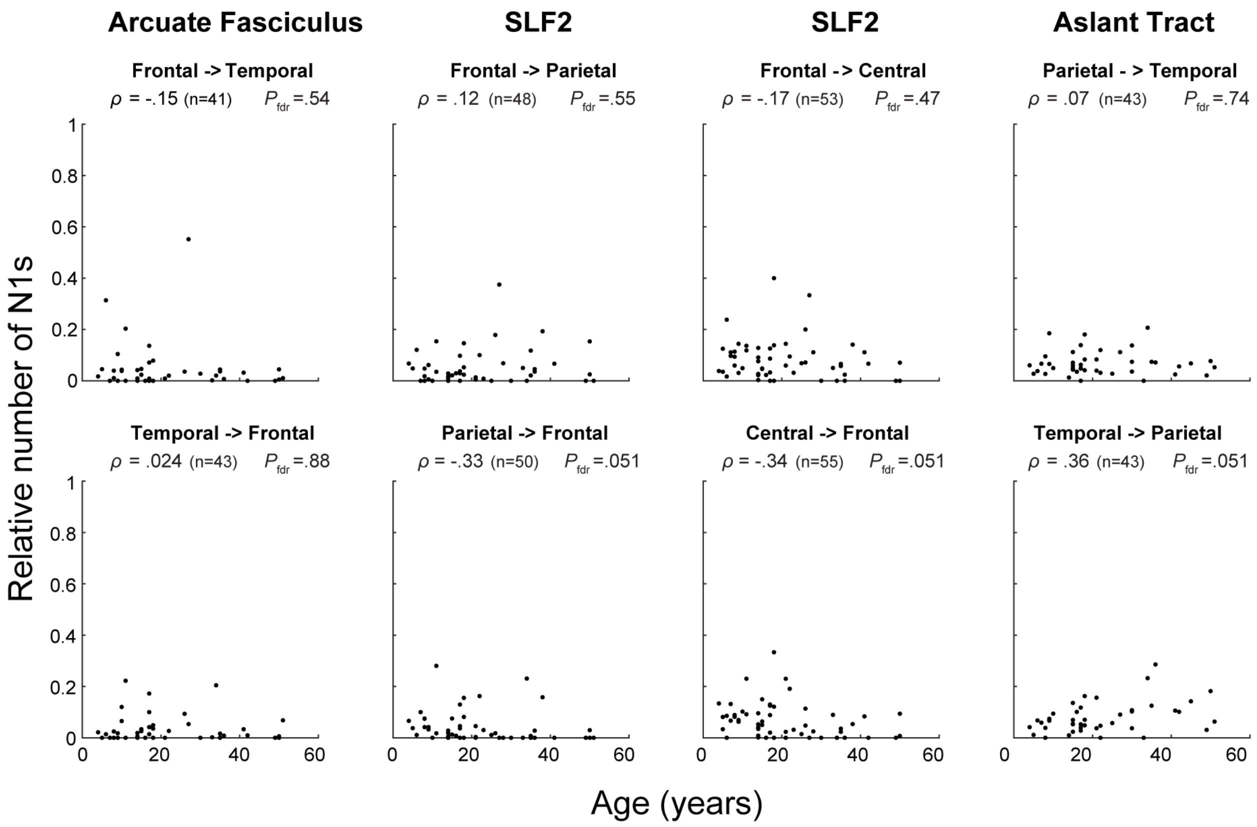

Supplementary Figure 2. Relation between age and relative number of N1s in long-range connections. Age (in years, x-axis) versus the relative number of N1s as a proportion of the total number of possible CCEPs within each connection (y-axis). Each dot represents one subject. The relative number of N1s per subject is determined by calculating - for each stimulus-pair with electrodes on the stimulus ROI - the ratio of N1s per the number of electrodes within the response ROI, and averaging over all these ratios. No significant relations between age and the relative number of CCEPs were observed (two-sided Spearman's ρ, $P_{FDR} < .05$). The FDR corrected P-values (from left-to-right and top-to-bottom) are: .54, .55, .47, .74, .88, .051, .051 and .051.

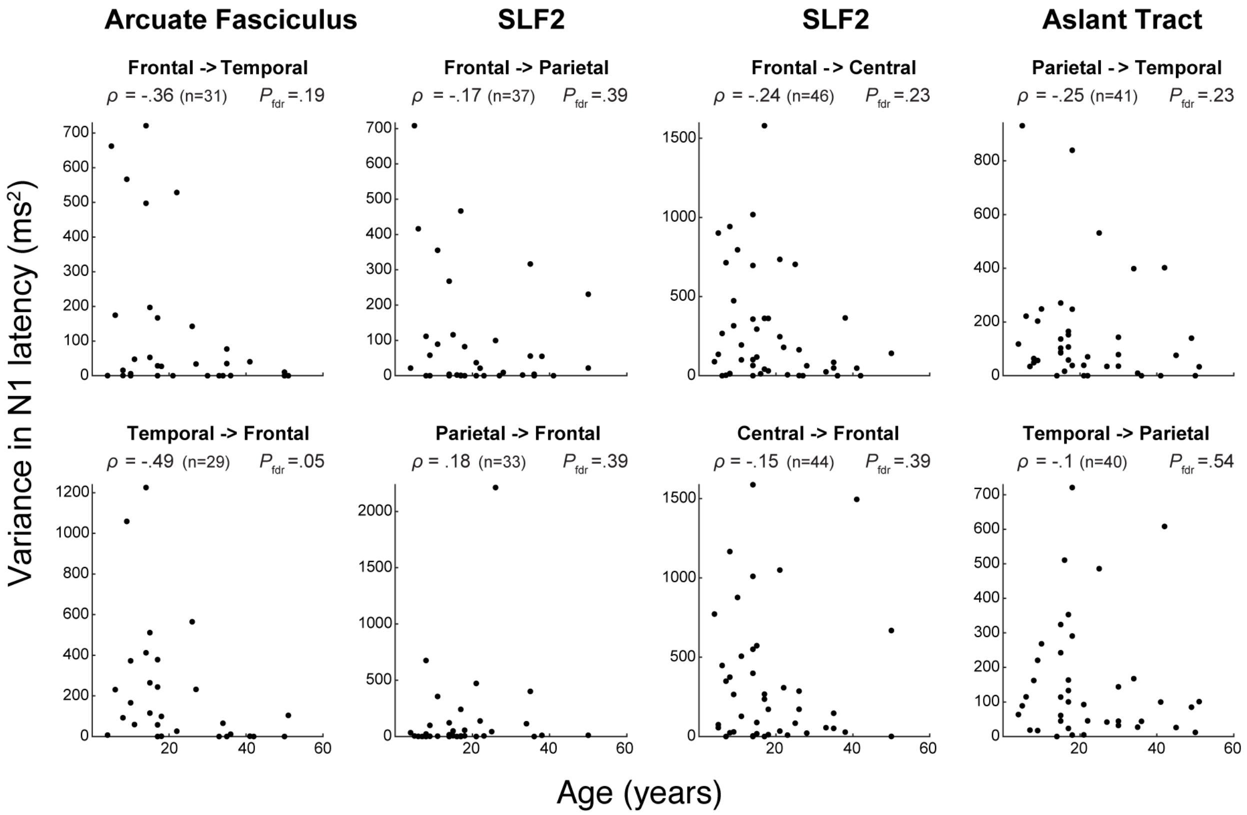

Supplementary Figure 3. Relation between age and variance in N1 peak latency in long-range connections. Age (in years, x-axis) versus the variance in N1 latency (in ms, y-axis). For each subject, we calculated the variance in latencies across connections in the same fiber pathway and tested whether increased variance relates to age. Each dot represents one subject. No significant relations between age and variance in latency were observed (two-sided Spearman's ρ, P_{FDR} < .05). The FDR corrected P-values (from left-to-right and top-to-bottom) are: .19, .39, .23, .23, .05, .39, .39 and .54.

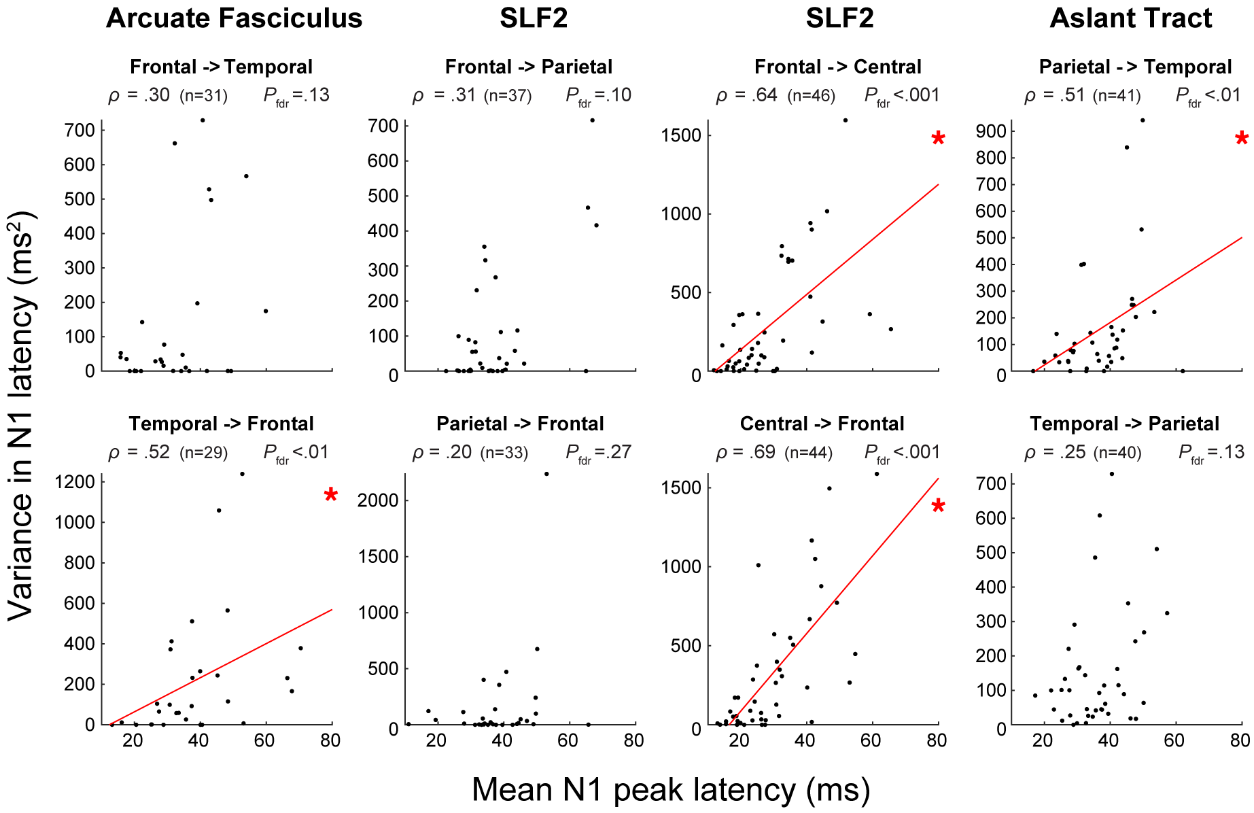

Supplementary Figure 4. Relation between mean N1 peak latency and variance in N1 peak latency in long-range connections. For each subject, we calculate the variance in N1 peak latencies across connections in the same fiber pathway and test whether increased latencies (in ms, x-axis) also have more variability (in ms, y-axis). Each dot represents the mean and variance in one subject. In 4 out of 8 long-range connections we observe a positive relation between the mean and variance in N1 latencies (two-sided Spearman's ρ, $P_{FDR} < .05$, red asterisk indicates significance). Showing that increased latency often relates to more variability. The FDR corrected P-values (from left-to-right and top-to-bottom) are: .13, .10, <.001, <.01, <.01, .27, <.001 and .13

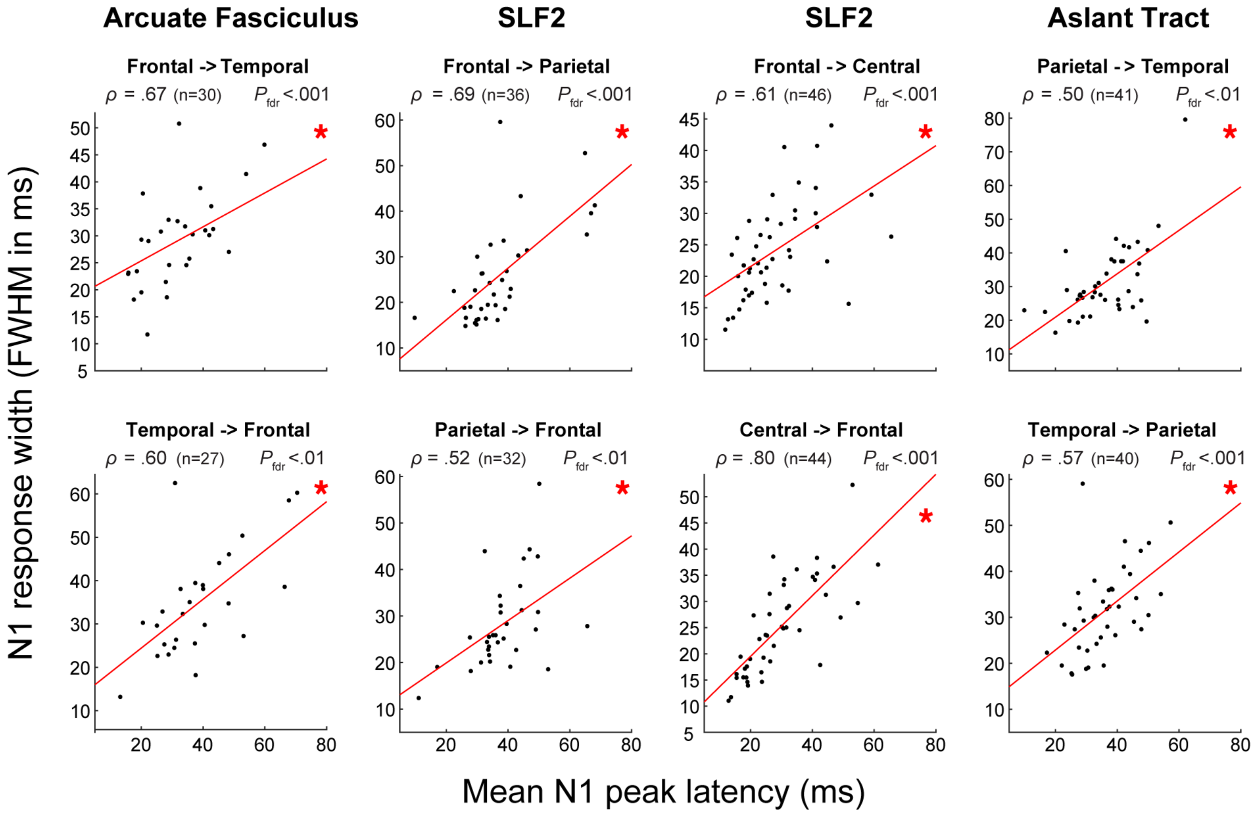

Supplementary Figure 5. Relation between mean N1 latency and N1 peak width. The relative temporal synchrony of the arriving signals in a measured electrode could be reflected in the width of the evoked potential. We therefore calculate the full width half max of the N1 peak, where the amplitude is 50% of the N1-peak amplitude (in ms, y-axis) and test whether this is related to the latency (in ms, x-axis). In all of the above connections, we observe a positive relation between the latency and the half N1 peak width (two-sided Spearman's ρ, $P_{FDR} < .05$, red asterisk indicates significance). The FDR corrected P-values (from left-to-right and top-to-bottom) are: <.001, <.001, <.001, <.01, <.01, <.01, <.001 and <.001.

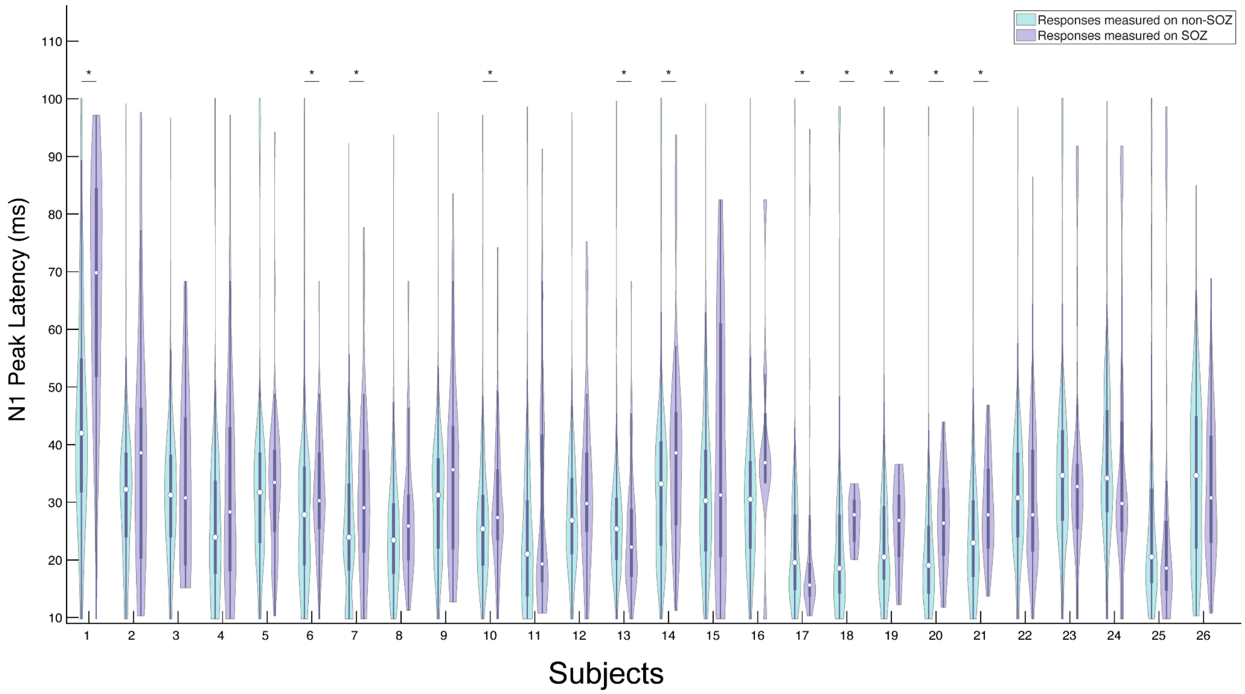

Supplementary Figure 6. The latency of N1 peaks measured on seizure onset zones (SOZ) or non-SOZs when stimulating elsewhere. For n=26 subjects this graph shows a box plot of the N1 latencies for electrodes measuring from the SOZ or non-SOZ areas. The central mark indicates the median latency, the bottom and top edges indicate 25th and 75th percentiles and the whiskers extend to extreme points excluding outliers (1.5 times more or less than the interquartile range). The asterisks display the subjects in whom a significant difference is found between latencies measured in SOZs and non-SOZs (two-sided Mann Whitney U test, $P_{FDR} < .05$). In 9 subjects, the latency is significantly increased in the SOZ. In 2 subjects, the latency is significantly decreased in the SOZ. The FDR corrected P-values for the tests between SOZ and non-SOZ (from left-to-right) are: <.001, .08, .87, .11, .40, .01, .02, .11, .10, <.01, .31, .10, <.01, <.01, .34, .11, <.001, <.01, .04, <.001, .01, .21, .33, .11, .23, and .23.

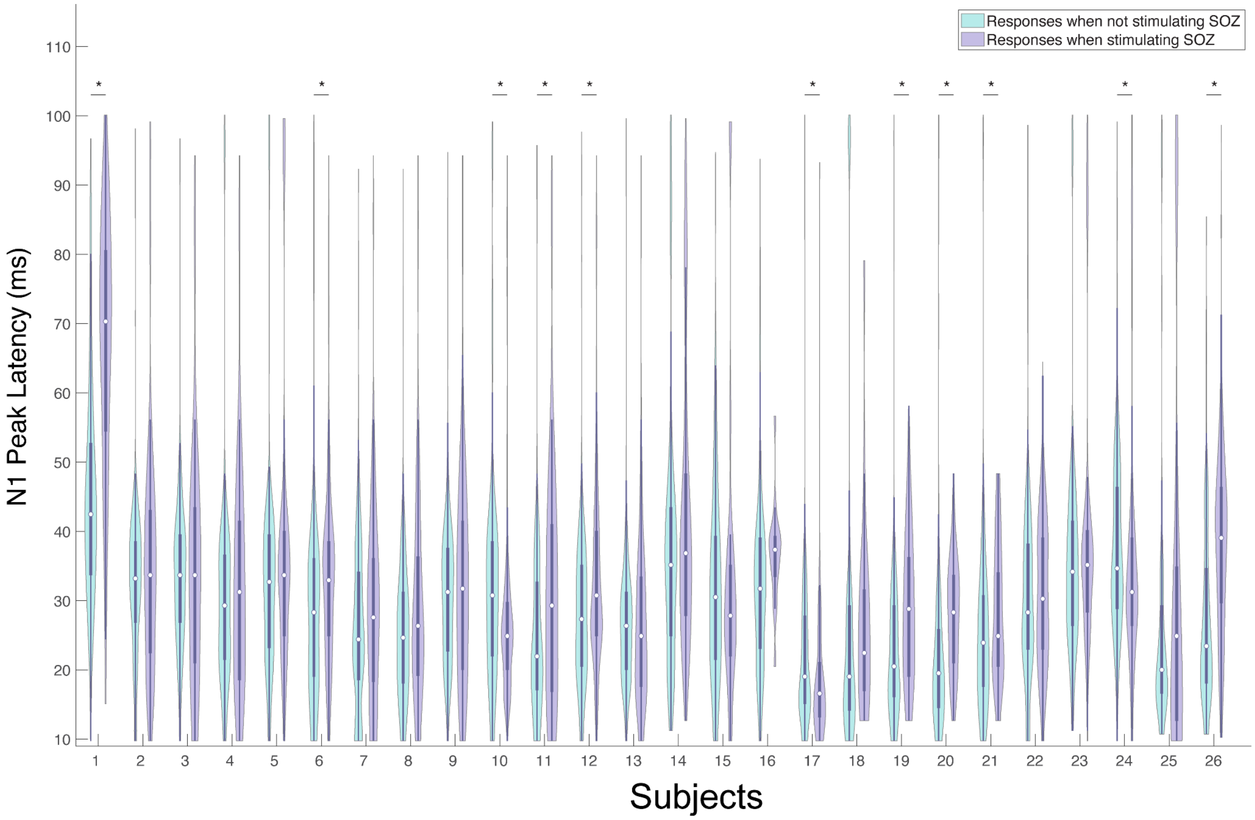

Supplementary Figure 7. The latency of N1 peaks in other electrodes when stimulating electrodes on seizure onset zones (SOZ) or on other areas (non-SOZs). For n=26 subjects this graph shows a box plot of N1 latencies after stimulating SOZ and non-SOZ. The central mark indicates the median, the bottom and top edges indicate 25th and 75th percentiles and the whiskers extend to extreme points excluding outliers (1.5 times more or less than the interquartile range). The asterisks display the subjects in whom a significant difference is found between latencies when stimulating SOZs and non-SOZs (two-sided Mann Whitney U test, $P_{FDR} < .05$). In 8 subjects, we find a significant increase in latency in response electrodes when the SOZ is stimulated. In 3 subjects, we find a significant decrease in latency in response electrodes when the SOZ is stimulated. The FDR corrected P-values for the tests between SOZ and non-SOZ (from left-to-right) are: <.001, .30, .97, .53, .07, <.001, .09, .09, .53, <.001, .01, .001, .33, .06, .74, .08, <.001, .20, <.001, <.001, .04, .47, .49, <.01, .81 and <.001.

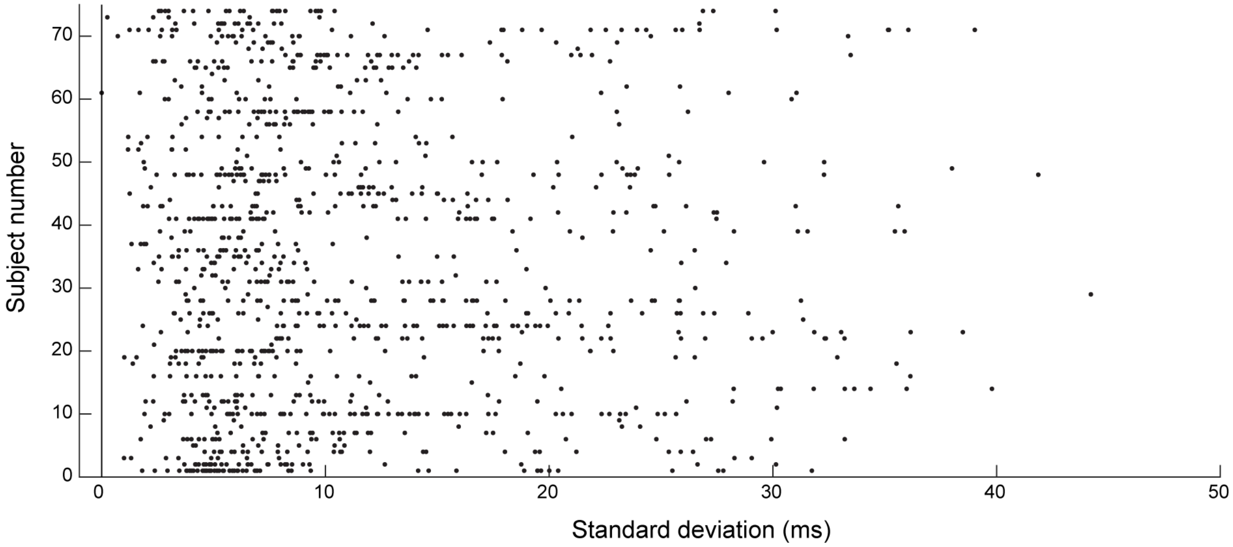

Supplementary Figure 8: Standard deviation across measured N1 latencies for each subject and each stimulated electrode pair. For each of the 74 subjects and stimulated electrode pair, we detected the N1 latencies and calculated the standard deviation across the N1 latencies when more than five N1 responses were detected (as with fewer responses, a standard deviation would not be robust). Each dot in a row represents a stimulated electrode pair in that subject and the x-axis indicates the standard deviation across the N1 latencies. If there would be volume conduction, N1 latency would be equal in all responses and the standard deviation would be zero, which is not the general case for these data.

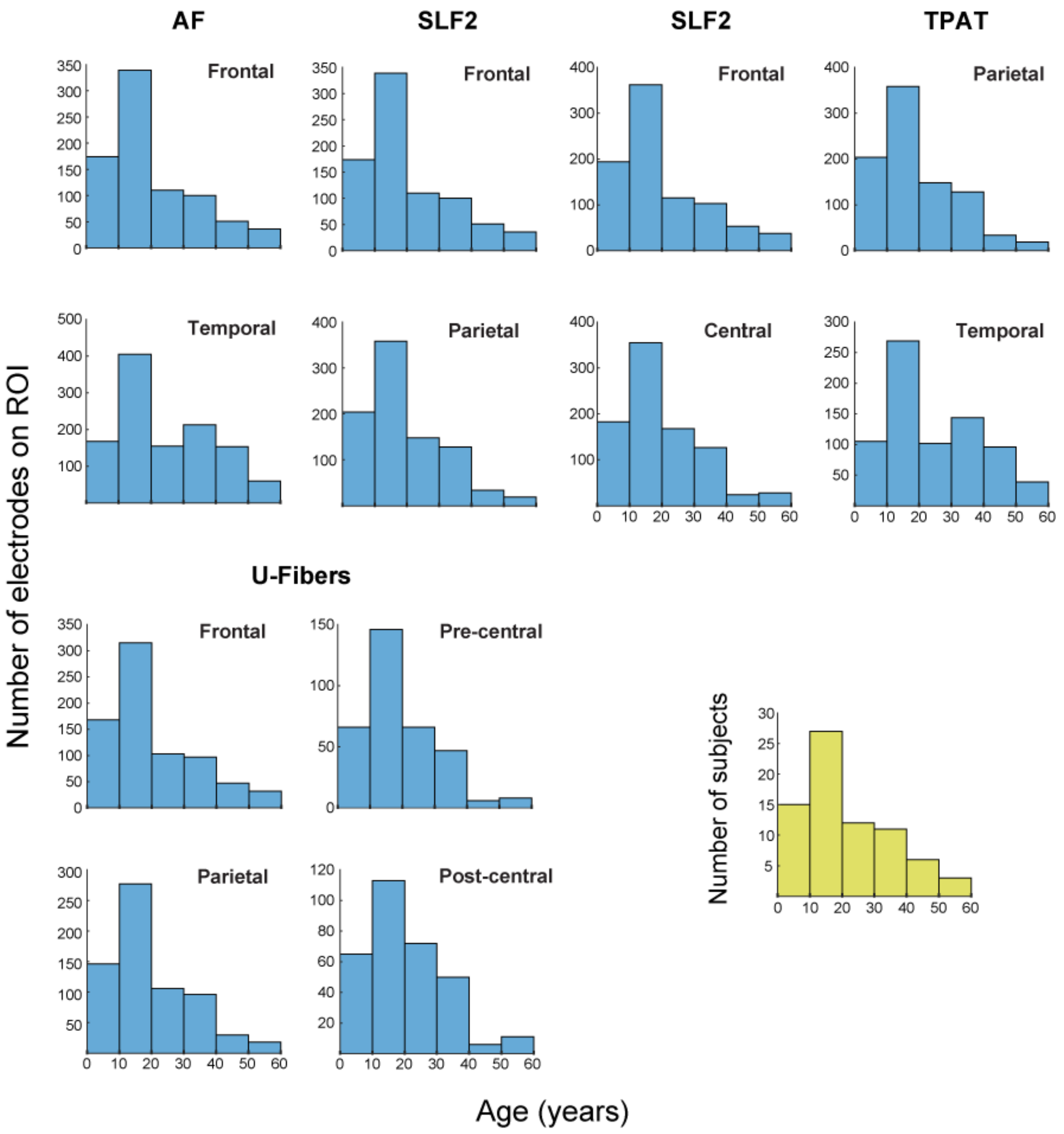

Supplementary Figure 9. Electrode distributions across ages for each tract and end-point region of interests (ROIs). The blue graphs show a histogram of the number of electrodes on top of the endpoint ROIs (y-axis) for each 10 years (x-axis). The yellow graph shows a histogram of the total number of participants for each 10 years (x-axis). The distribution of electrodes over age follows the distribution of subjects over age, implying that the electrodes are relatively uniformly distributed across the age ranges.

Tract	End-point region	Destrieux regions	Number of subjects with coverage	Total #electrodes
Arcuate Fasciculus	Frontal	G_front_inf-Opercular, G_front_inf-Triangul, G_front_middle, S_front_inf, S_front_middle	58	810
	Temporal	G_temp_sup-Lateral, G_temp_sup-Plan_tempo, G_temporal_inf, G_temporal_middle, S_temporal_inf, S_temporal_sup	54	1154
SLF2	Frontal	G_front_inf-Opercular, G_front_inf-Triangul, G_front_middle, S_front_inf, S_front_middle	58	810
	Parietal	G_pariet_inf-Angular, G_pariet_inf-Supramar, G_parietal_sup, S_intrapariet&P_trans	66	891
SLF2	Frontal	G_front_inf-Opercular, G_front_inf-Triangul, G_front_middle, S_front_inf, S_front_middle, S_front_sup	58	865
	Central	G&S_paracentral, G&S_subcentral, G_and_S_subcentral, G_postcentral, G_precentral, S_central	68	885
TPAT	Parietal	G_pariet_inf-Angular, G_pariet_inf-Supramar, G_parietal_sup, S_intrapariet&P_trans	66	891
	Temporal	G_temp_sup-G_T_transv, G_temporal_inf, G_temporal_middle, S_oc-temp_lat, S_temporal_sup, S_temporal_transverse	47	755
U-Fibers	Frontal	G_front_inf-Opercular, G_front_inf-Triangul, G_front_middle	58	762
	Parietal	G_pariet_inf-Angular, G_pariet_inf-Supramar	65	673
	Pre-central	G_precentral	54	339
	Post-central	G_postcentral	54	317

Supplementary Table 1. Overview of number of electrode positions in different brain regions. Electrodes were assigned to several brain regions based on the label from the Destrieux atlas in Freesurfer.

Reference	Modality/Method	N	Age (yr)	Latency
Allison et al. (1984)<br><br>Developmental and aging changes in somatosensory, auditory and visual evoked potentials	- EEG<br>- Auditory: clicks<br>- Visual: checkerboard	286	4-17<br>18-95	Auditory (fig 4):<br>- P2-P9<br>(age 4-17) = N.S.<br>(age 18-95) = ~ +0.003-0.004 ms/yr<br><br>Visual (fig 5):<br>- P60 and N75<br>(age 4-95) = ~ +0.035-0.045 ms/yr<br>- P100<br>(age 4-20/30) = ~ -0.4 ms/yr<br>(age 60 - 95) = ~ +0.3 ms/yr<br>- N145<br>(age 4-20/30) = ~ -0.9 ms/yr<br>(age 60 - 95) = ~ +0.6 ms/yr
Armstrong et al. (1991)<br><br>Visual Evoked Magnetic Fields to Flash and Pattern in 100 Normal Subjects	- MEG<br>- Visual: checkerboard and flashes	100	15-87	Visual (Fig 3 and 4):<br>- P100M<br>(age 15-54) = ~ +0.12 ms/yr<br>(age 55-87) = ~ +1.40 ms/yr<br>- P2M<br>(age 15-43) = ~ -0.7 ms/yr<br>(age 44-87) = ~ +0.93 ms/yr
Coffara et al. (2021)<br><br>Development of the Visual White Matter Pathways Mediates Development of Electrophysiological Responses in Visual Cortex	- MEG<br>- Visual: image w. word	46	7-12	Visual (fig 4):<br>- M100<br>(age 7-17) = ~ -3.4 ms/yr
Emmerson-Hanover et al. (1994)<br><br>Pattern reversal evoked potentials gender differences and age-related changes in amplitude and latency	- EEG<br>- Visual: checkerboard	406	6-80	Visual:<br>- P50<br>(females age 6-20) = ~ -0.38 ms/yr<br>(males age 6-20) = ~ +0.12 ms/yr<br>(age 21-80) = +0.19 ms/yr<br><br>- P70<br>(age 6-80) = +0.12 ms/yr
Mahajan et al. (2012)<br><br>Maturation of Visual Evoked Potentials across Adolescence.	- EEG<br>- Visual: checkerboard	100	10-25	Visual:<br>- N75:<br>(age 10-25) = N.S.<br>- P100:<br>(age 10-25) = N.S.<br>- N135:<br>(age 10-25) = -0.44-0.77 ms/yr

Polich et al. (1997)<br><br>EEG and ERP assessment of normal aging	- EEG<br>- Visual: checkerboard<br>- Auditory: tones	120	20-~90	Visual (fig 8):<br>- P3<br>(age 20-90) = ~ +0.5 ms/yr<br><br>Auditory (fig 8):<br>- P3<br>(age 20-90) = ~ +1 ms/yr
Shaw et al. (1980)<br><br>Age-dependent changes in the latency of the pattern visual evoked potential	- EEG<br>- Visual: checkerboards	71	16-72	Visual (fig 1):<br>- P100 (low luminance)<br>(age 16-40) = -0.077 ms/y<br>(age 40-72) = +0.158 ms/y
Sokol et al. (1981)<br><br>Age-related changes in the latency of the visual evoked potential: Influences of check size	- EEG<br>- Visual: checkerboard	125	13-82	Visual:<br>- P1<br>check size 12:<br>(age 13-82) = +0.26 ms/yr<br>check size 48:<br>(age 13-82) = +0.14 ms/yr
Stockard et al. (1979)<br><br>Visually Evoked Potentials to Electronic Pattern Reversal Latency Variations with Gender, Age, and Technical Factors	- EEG<br>- Visual: checkerboard	100	13-67	Visual:<br>- P1<br>(age 13-67) = N.S.
Tobimatsu et al. (1993)<br><br>Age-related changes in pattern visual evoked potentials: differential effects of luminance, contrast and check size	- EEG<br>- Visual: checkerboard	105	19-84	Visual (fig 1, 3):<br>- P100<br>(age 19-45) = ~ -0.25 ms/yr<br>(age 45-84) = ~ +0.5 ms/yr
Van Dinteren et al. (2014)<br><br>P300 development across the lifespan: a systematic review and meta-analysis.	- EEG<br>- Auditory: tones	meta analysis: 2811<br><br>cross sectional data: 1964	meta analysis: 4-95<br><br>cross sectional data: 6-87	Auditory (fig 3, 4):<br>- P300<br>(age 4-22) = ~ -5 ms/yr<br>(age 22-90) = ~ +1 ms/yr

Supplementary Table 2. Short overview of studies that investigate evoked potentials over age. Latency increases are marked in red, decreases in blue, no significant changes in green.
