# Supplementary material for: Developmental trajectory of transmission speed in the human brain
Source: Nat Neurosci. 2023 Mar 9;26(4):537–41. doi: 10.1038/s41593-023-01272-0 (PMC10076215; doi:10.1038/s41593-023-01272-0)
Supplement: Supplementary file 2 — Reporting Summary [file 41593_2023_1272_MOESM2_ESM.pdf]

nature portfolio

Corresponding author(s): Dora Hermes

Last updated by author(s): 2022/03/22

Reporting Summary

Nature Portfolio wishes to improve the reproducibility of the work that we publish. This form provides structure for consistency and transparency in reporting. For further information on Nature Portfolio policies, see our Editorial Policies and the Editorial Policy Checklist.

Statistics

For all statistical analyses, confirm that the following items are present in the figure legend, table legend, main text, or Methods section.

n/a	Confirmed	
☐	☒	The exact sample size (*n*) for each experimental group/condition, given as a discrete number and unit of measurement
☐	☒	A statement on whether measurements were taken from distinct samples or whether the same sample was measured repeatedly
☐	☒	The statistical test(s) used AND whether they are one- or two-sided<br>*Only common tests should be described solely by name; describe more complex techniques in the Methods section.*
☐	☒	A description of all covariates tested
☐	☒	A description of any assumptions or corrections, such as tests of normality and adjustment for multiple comparisons
☐	☒	A full description of the statistical parameters including central tendency (e.g. means) or other basic estimates (e.g. regression coefficient) AND variation (e.g. standard deviation) or associated estimates of uncertainty (e.g. confidence intervals)
☐	☒	For null hypothesis testing, the test statistic (e.g. *F*, *t*, *r*) with confidence intervals, effect sizes, degrees of freedom and *P* value noted<br>*Give P values as exact values whenever suitable.*
☒	☐	For Bayesian analysis, information on the choice of priors and Markov chain Monte Carlo settings
☒	☐	For hierarchical and complex designs, identification of the appropriate level for tests and full reporting of outcomes
☐	☒	Estimates of effect sizes (e.g. Cohen's *d*, Pearson's *r*), indicating how they were calculated

Our web collection on statistics for biologists contains articles on many of the points above.

Software and code

Policy information about availability of computer code

Data collection	No software was used for data collection.
Data analysis	For segmenting the imaging data we used Freesurfer V6.0. We used ANTs registration in lead-dbs V1.6.3 to register white matter atlases to the native space of each subject. The Matlab code to analyze the data and generate all figures of this manuscript is available on GitHub: https://github.com/MultimodalNeuroimagingLab/mnl_ccepBids and is tested with Matlab versions 2021a and 2022a.

For manuscripts utilizing custom algorithms or software that are central to the research but not yet described in published literature, software must be made available to editors and reviewers. We strongly encourage code deposition in a community repository (e.g. GitHub). See the Nature Portfolio guidelines for submitting code & software for further information.

Data

Policy information about availability of data

All manuscripts must include a data availability statement. This statement should provide the following information, where applicable:

- Accession codes, unique identifiers, or web links for publicly available datasets
- A description of any restrictions on data availability
- For clinical datasets or third party data, please ensure that the statement adheres to our policy

The data that support the findings of this study are being made available in BIDS format on OpenNeuro: https://openneuro.org/datasets/ds004080. Identifiers are removed from the data and electrode positions with anatomical Destrieux atlas labels are shared in MNI152 space to remove the need for sharing MRI scans. Atlases of white matter tracts were defined based on the population-averaged tractography atlasses HCP1065 (AF, SLF, TPAT) and HCP842 (U-fibers): https://brain.labsolver.org/hcp_trk_atlas.

Field-specific reporting

Please select the one below that is the best fit for your research. If you are not sure, read the appropriate sections before making your selection.

☒ Life sciences ☐ Behavioural & social sciences ☐ Ecological, evolutionary & environmental sciences

For a reference copy of the document with all sections, see nature.com/documents/nr-reporting-summary-flat.pdf

Life sciences study design

All studies must disclose on these points even when the disclosure is negative.

Sample size	No statistical methods were used to pre-determine sample sizes and we included all subjects who underwent Single Pulse Electrical Stimulation (SPES) for clinical purposes during the intracranial grid monitoring period between 2012 and 2020 and met inclusion criteria, resulting in 74 subjects included in the study. For these subjects, we analyzed connections where at least 20 subjects had electrodes on both endpoints, and exclude connections to regions without sufficient electrode coverage for across-subject correlations, such as the occipital lobe. A retrospective power analysis with an expected Pearson correlation of .60, a=.05, and desired power of 80%, tells us a sample size of at least 19 is desirable.
Data exclusions	Subjects who underwent epilepsy surgery in the UMC Utrecht between 2008 and 2020, underwent single pulse electrical stimulation and did not have large structural abnormalities were included in analyses. Standard, pre-established criteria were used to review iEEG data: electrodes and data segments with excessive noise were annotated in the data (following BIDS structure) and are excluded from analyses. Stimulation pairs that introduced baseline offsets on many measured channels were additionally excluded from all analyses.
Replication	We did not include multiple groups to replicate results.
Randomization	There were no experimental groups.
Blinding	Subjects were not assigned to groups.

Reporting for specific materials, systems and methods

We require information from authors about some types of materials, experimental systems and methods used in many studies. Here, indicate whether each material, system or method listed is relevant to your study. If you are not sure if a list item applies to your research, read the appropriate section before selecting a response.

Materials & experimental systems

n/a	Involved in the study	
☒	☐	Antibodies
☒	☐	Eukaryotic cell lines
☒	☐	Palaeontology and archaeology
☒	☐	Animals and other organisms
☐	☒	Human research participants
☒	☐	Clinical data
☒	☐	Dual use research of concern

Methods

n/a	Involved in the study	
☒	☐	ChIP-seq
☒	☐	Flow cytometry
☒	☐	MRI-based neuroimaging

Human research participants

Policy information about studies involving human research participants

Population characteristics	This study includes 74 subjects who underwent epilepsy surgery in the UMC Utrecht between 2008 and 2020, the median age was 17 years (range 4-51 years), there were 38 females.
Recruitment	The study was partially retrospective (before 2018), with IRB permission. After 2018, subjects undergoing epilepsy monitoring and single pulse electrical stimulation for clinical purposes were asked for informed consent to include their data for research purposes. Recruitment may therefore include biases from this population, which we address in the manuscript.
Ethics oversight	Medical Research Ethical Committee from the UMC Utrecht

Note that full information on the approval of the study protocol must also be provided in the manuscript.
